# Supplementary material for: Imaging of Antiferroelectric Dark Modes in an Inverted Plasmonic Lattice
Source: ACS Nano. 2023 Apr 24;17(9):8123–32. doi: 10.1021/acsnano.2c11016 (PMC10173685; doi:10.1021/acsnano.2c11016)
Supplement: Supplementary file 1 — nn2c11016_si_001.pdf [file nn2c11016_si_001.pdf]

Supporting information for:

Imaging of Antiferroelectric Dark Modes in an Inverted Plasmonic Lattice

Javier Rodríguez-Álvarez[1,2,], Amílcar Labarta[1,2], Juan Carlos Idrobo[3], Rossana Dell'Anna[4], Alessandro Cian[4], Damiano Giubertoni[4], Xavier Borrisé[5], Albert Guerrero[6], Francesc Perez-Murano[6], Arantxa Fraile Rodríguez[1,2] and Xavier Batlle[1,2]*

[1] Departament de Física de la Matèria Condensada, Universitat de Barcelona, 08028 Barcelona, Spain

[2] Institut de Nanociència i Nanotecnologia (IN2UB), Universitat de Barcelona, 08028, Spain

[3] Materials Science and Engineering Department, University of Washington, Seattle, WA, 98195, USA

[4] Sensors & Devices Center, FBK - Bruno Kessler Foundation, via Sommarive, 18, Povo, TN 38123 Italy

[5] Catalan Institute of Nanoscience and Nanotechnology (ICN2), CSIC and BIST, Campus UAB, Bellaterra, 08193 Barcelona, Spain

[6] Institut de Microelectrónica de Barcelona (IMB-CNM, CSIC), Bellaterra, 08193, Spain

E-mail: javier.rodriguez@ub.edu

- **S1.** Transversal electric field distribution for the SLR at 1.57 eV.
- **S2.** Simulated electric field and charge distributions for a threesome of slits.
- **S3.** Simulated electric field and charge distributions for the simplest local dark mode of the inverted honeycomb lattice.
- **S4.** Profiles of the EELS signal and the simulated electric field along the slits for the antiferroelectric dark modes.

- **S5.** Array of the magnetic dipoles over the structure used to simulate antiferroelectric dark modes.

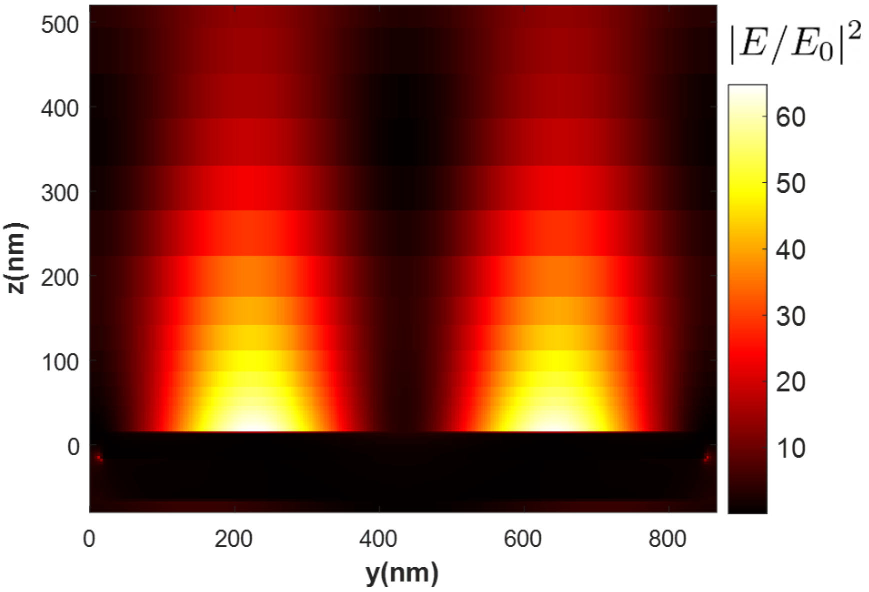

Figure S1. Simulated electric field for the SLR at 1.57 eV. The zy plane, transversal to the structure, shows the strong out-of-plane electric field of a unit cell. Each one of the two hotspots corresponds to transversal cuts to the ring shown in Fig. 3i. z = 0 nm corresponds to the center of the metallic gold layer.

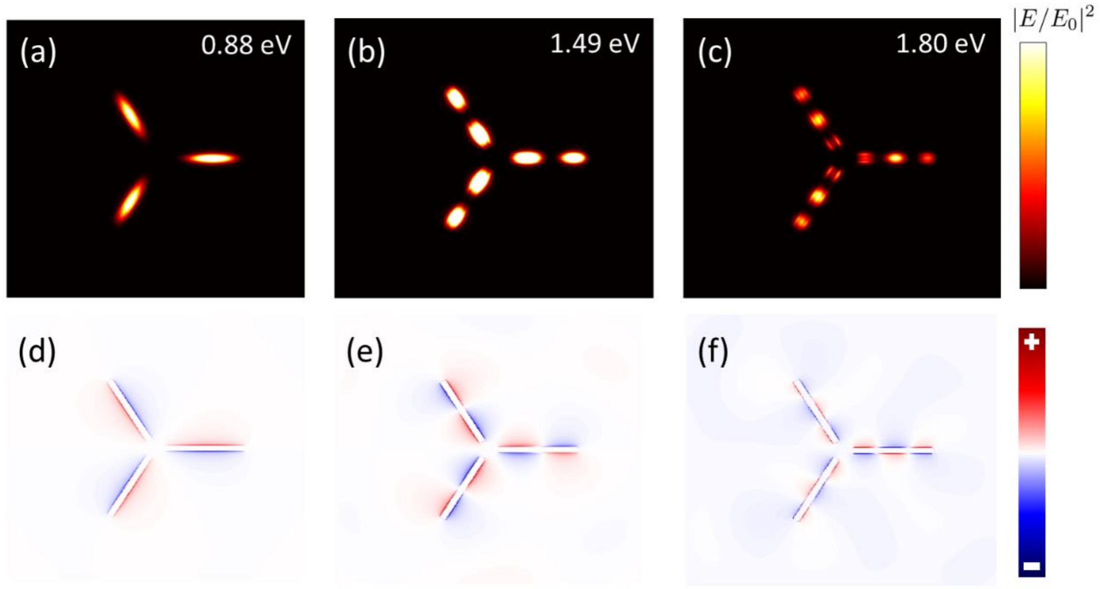

Figure S2. (a, b, c) Electric field and (d, e, f) charge distributions for the dark modes with the lowest energies in the three slits system. The plasmonic modes have been excited by placing a magnetic dipole 50 nm above the vertex of the slits.

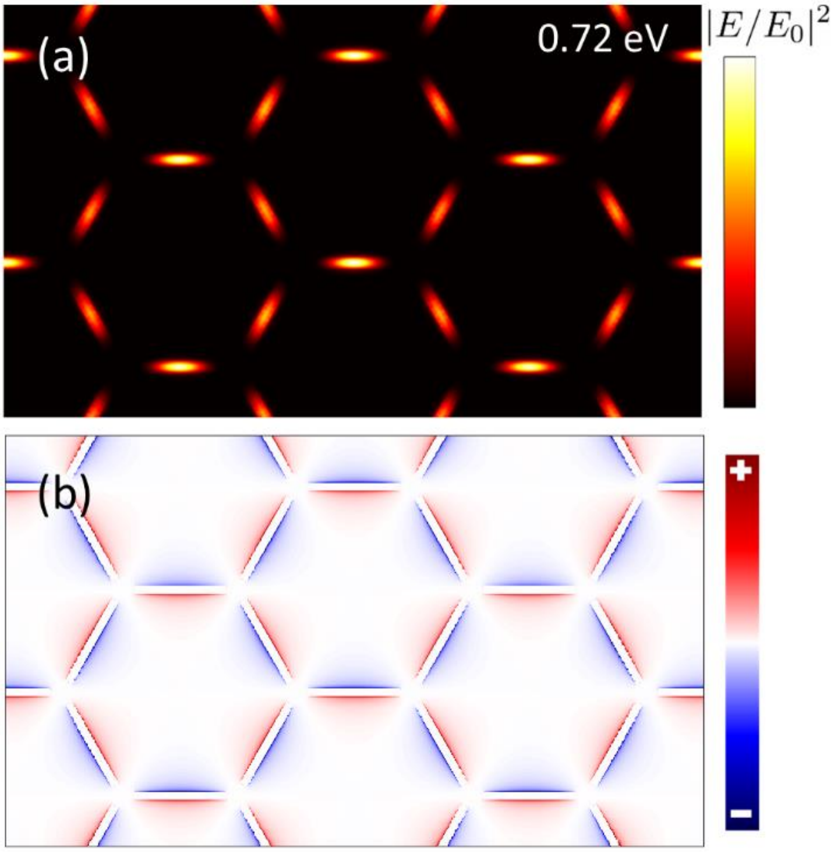

Figure S3. (a) Electric field and (c) charge distributions for the simplest local dark mode. Note that despite the non-zero dipole moment of the slits, the net moment is zero if the whole unit cell is considered. This plasmonic mode has been excited by placing an array of magnetic dipoles 50 nm above the vertices of the hexagons coincident with the Bravais lattice.

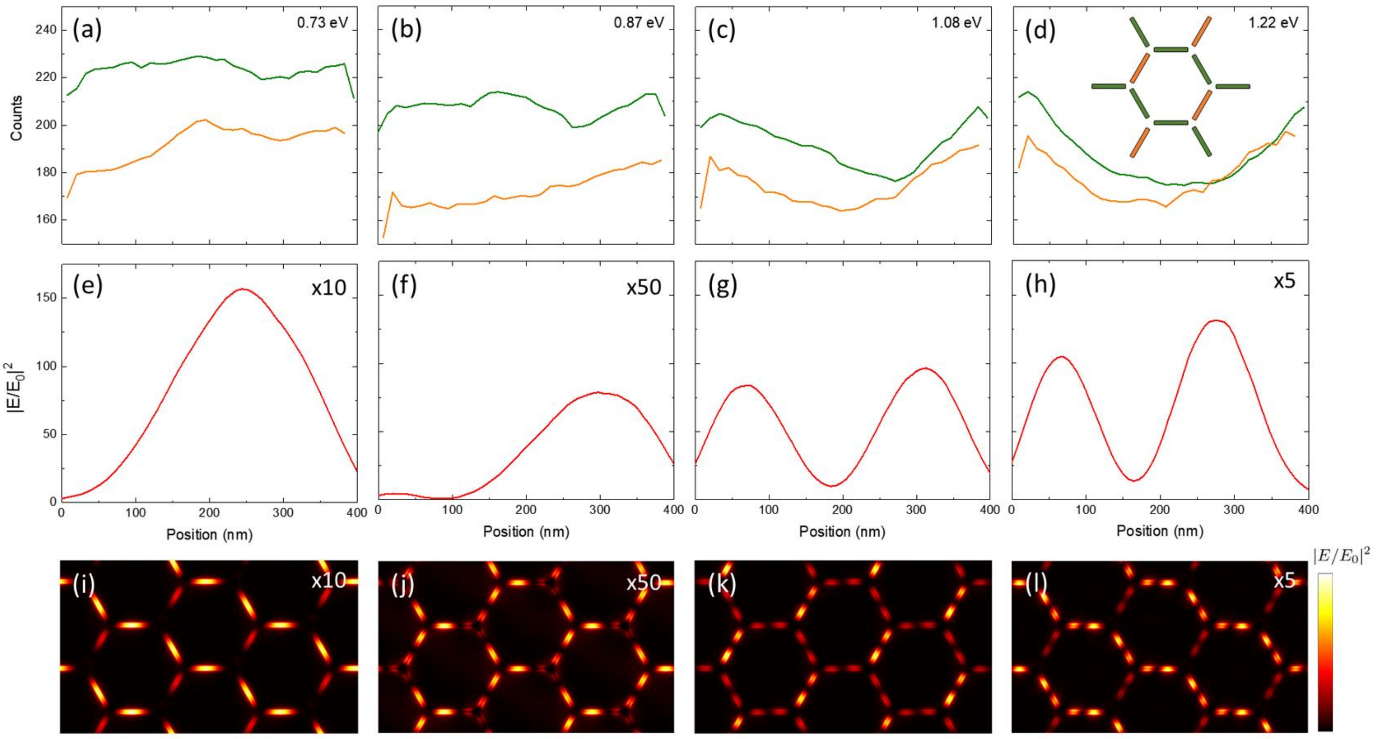

Figure S4. (a,b,c,d) EELS intensity profile along the slits for four energies corresponding to the range where antiferroelectric dark modes are detected by EELS. The green and orange curves depict the average intensity of the EELS signal as a function of the position for the two subsets of slits depicted in the inset. (e,f,g,h) Simulated intensity of the electric-field profile along a slit for each energy, computed averaging the three variants of the modes shown in panels (i,j,k,l). The charge intensity has been multiplied by a factor indicated in the top-right side of the panels for an easier comparison between resonances. (i,j,k,l) Near-field distributions corresponding to one of the three variants (equivalent by three-fold symmetry) for each energy. Note that the energies correspond to those of the EELS color maps shown in Figure 6.

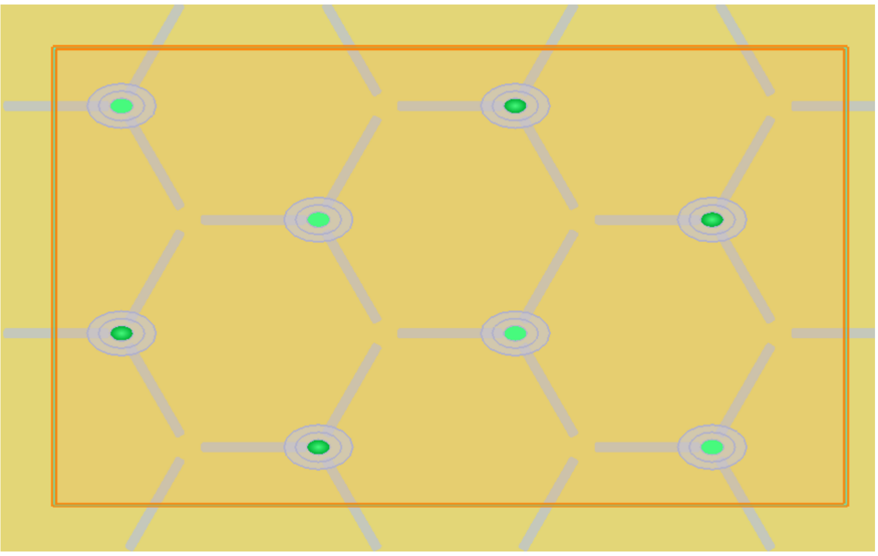

Figure S5. Schematics of the unit cell for the simulations of the antiferroelectric modes where the excitation source is an array of magnetic dipoles. There are two subsets of magnetic dipoles (lighter and darker green circles) in phase opposition with respect to each other. Note the simulation region denoted by an orange rectangle. The magnetic dipoles are placed 50 nm above the vertices of the hexagons that are coincident with the Bravais lattice.
